# Frontal neural metabolite changes in schizophrenia and their association with cognitive control: A systematic review

**DOI:** 10.1016/j.neubiorev.2021.11.010

**Published:** 2022-01

**Authors:** Bradley J. Dixon, Jyothika Kumar, Claudia Danielmeier

**Affiliations:** a University of Nottingham, School of Psychology, United Kingdom; b Cambridgeshire Community Services NHS Trust, United Kingdom

**Keywords:** Cognitive control, Cognition, Frontal, Negative symptoms, Schizophrenia, Metabolite, GABA, Glutamate, Glx, Magnetic resonance spectroscopy, MRS, magnetic resonance spectroscopy

## Abstract

A large proportion of patients with schizophrenia exhibit deficits in cognitive control functions including working memory, processing speed and inhibitory control, which have been associated with frontal brain areas. In this systematic review, we investigated differences between chronic schizophrenia patients, first-episode (FEP) patients and healthy control groups in the neurometabolite levels of GABA, glutamate, glutamine and Glx in frontal brain areas. Additionally, we reviewed correlations between cognitive control functions or negative symptoms and these neurometabolite levels. Several studies reported decreased GABA or glutamate concentrations in frontal lobe areas, particularly in chronic schizophrenia patients, while the results were mixed for FEP patients. Working memory performance and prediction errors have been associated with frontal GABA and glutamate levels, and processing speed with frontomedial GABA levels in chronic patients. The relationship between metabolites and negative symptom severity was somewhat inconsistent. Future studies should take the participants' age, medication status or responsivity, disease stage and precise anatomical location of the voxel into account when comparing neurometabolite levels between schizophrenia patients and healthy controls.

## Highlights

• GABA levels are decreased in medial frontal brain areas of schizophrenia patients.
• Glutamate levels are lower in medial and lateral frontal areas in chronic patients.
• Working memory performance is associated with frontal GABA and Glu.
• Prediction errors are associated Glu and medial frontal GABA.
• Processing speed correlates with medial frontal GABA levels.

## 1 Introduction

Individuals with schizophrenia (SZ) are not just affected by positive and negative, but also by cognitive symptoms (e.g., Barch and Ceaser, 2012; Guo et al., 2019; Sheffield et al., 2019; Storchak et al., 2021). About 75–80 % of schizophrenia patients experience cognitive deficits (Palmer et al., 2009). Indeed, it has been suggested that schizophrenia should be viewed as a cognitive illness (Kahn and Keefe, 2013; Sheffield et al., 2019) The successful treatment of cognitive impairments in individuals with schizophrenia predicts socio-occupational functioning, e.g. if a patient is capable of returning to work or school within 9 months of the onset of the illness (Nuechterlein et al., 2011). There have been reported deficits in patients on tests of memory (e.g. Guo et al., 2019; Mohamed et al., 1999), attentional processes (e.g. Hoonakker et al., 2017; Saykin et al., 1994) and executive functioning (e.g. Hutton and Kennard, 1998; Lim et al., 2021; Storchak et al., 2021). Successful goal-directed actions require adequate planning, and subsequent adjustment of behaviour determined by acquiring task-specific information and ignoring interfering stimuli. Barch and Ceaser (2012) suggested that modulations in cognitive control could be pivotal for several different cognitive impairments due to deficits in goal maintenance in schizophrenia patients. Cognitive control has been functionally linked with the frontal lobe (Ullsperger et al., 2014), with localised regions being intrinsically associated with functionally different aspects of cognitive control (Ridderinkhof et al., 2004). Selective attention and working memory are cognitive functions that are closely linked to cognitive control. Individuals with schizophrenia show profound deficits in both cognitive functions (Guo et al., 2019). Additionally, Ullsperger (2006) summarized deficits in performance monitoring that have been observed in schizophrenia patients and are associated with modulated functions in the posterior medial frontal cortex (pMFC). One aim of the current review is to investigate if there are systematic links between modulations in these cognitive control functions and neurometabolite changes in the frontal lobes of individuals with schizophrenia. As a first step, we will review reported baseline differences in frontal metabolite levels between individuals with schizophrenia (chronic patients and first-episode patients separately) and healthy controls, before we report correlations between these metabolites and cognitive functions or symptom severity, respectively.

Historically, the driving factor behind the symptoms and impairments of schizophrenia were attributed to the role of dopamine. Traditional antipsychotic treatments for schizophrenia rely on the blocking of D2 dopamine receptors, which are efficacious in diminishing prominent positive symptoms, but fail to treat many of the more debilitating negative and cognitive symptoms (Seeman, 2002; Lieberman et al., 2005). The inefficacy of treatment therefore suggested the involvement of other neurotransmitter systems. Recent studies have suggested the glutamatergic system and related metabolites may offer a more holistic explanation to the persistence of cognitive impairment (Coyle, 2006; cf. Reddy-Thootkur et al., 2020). A proposed pathway suggests the hypofunction of the N-Methyl-D-Aspartate Glutamate receptor (NMDAR), critical in the production, release and reabsorption of neural metabolites including glutamate (Glu), glutamine (Gln) and gamma-Aminobutyric acid (GABA; Coyle, 2006). Pharmacological intervention studies have shown that the antagonism of the NMDAR pathway using ketamine, phencyclidine (PCP), exhibits symptoms of schizophrenia in healthy participants (Lahti et al., 1995). In comparison, dopamine agonism has been appreciated to only successfully model the positive symptoms of schizophrenia (Beck et al., 2020; Krystal et al., 2005). The potential functional modulation in the glutamatergic system remains intrinsically relevant here, as it has been shown that the modulation of these neural metabolites results in modulations of performance in several cognitive tasks (Thomas et al., 2017; Dauvermann et al., 2017). In humans, in vivo measurements of neural metabolites can be performed with ^1^H-Magnetic Resonance Spectroscopy (MRS).

### 1.1 ^1^H-magnetic resonance spectroscopy

^1^H-Magnetic Resonance Spectroscopy (MRS) is a non-invasive in vivo imaging technique, capable to provide measurements of metabolite concentrations in the human and animal brain. Advancements in hardware, and development of specific pulse sequences, have improved the efficacy of measurements of Glu, Gln and GABA. Historically, Glu and Gln were reported as a single measurement (Glx) as the magnet field strength was ineffective in separating the signal from the two metabolites. Furthermore, pulse sequences have been developed to enhance the signal from GABA to ensure that measurements taken in vivo are as reliable as possible (Lally et al., 2016).

### 1.2 Current review

The hypothesized action of glutamatergic metabolites as an explanation for the development of schizophrenia symptoms is promising, and yet has generated inconclusive results across studies (Dauvermann et al., 2017). The short-term result of differences in metabolite levels may well have different manifestations than prolonged exposure. This could result in differences in the severity of symptoms between chronic patients who have lived with the condition for a prolonged period of time, and patients exhibiting symptoms for the first time (Coyle, 2006; Dauvermann et al., 2017). Therefore, we will summarize the spectroscopy findings separately for chronic and first-episode (FEP) patients. Additionally, newer studies that utilize higher field strength magnetic resonance scanners and advanced imaging techniques may help to elucidate consistencies in metabolite levels in association with behavioral patterns (Lally et al., 2016). In this review, we summarize results from MRS studies performed on both chronic and FEP SZ patients and healthy controls in the frontal lobe to describe differences in frontal GABA, Glu, Gln and Glx concentrations between groups. We then focus on studies that reported associations between these neurometabolite concentrations in frontal lobe regions and both symptom severity and cognitive control functions.

## 2 Methods

This systematic review was conducted in accordance with the Preferred Reporting Items for Systematic Reviews and Meta-Analyses (PRISMA; Moher et al., 2009) protocols. The intention and outline of the review was registered with PROSPERO (Page et al., 2018; registration number: CRD42020222884; [https://www.crd.york.ac.uk/prospero/display_record.php?RecordID=222884]). Only articles that were published in English language up to, and including, 2020 were included in the review.

A PubMed search was conducted on 20th June 2020 using the following terms:

(mrs OR spectroscopy OR proton) AND (glutamate OR glutamine OR GABA* OR Gamma* OR γ-amino*) AND (schizophren* OR psychosis OR psychotic) AND (front* OR med*) AND (brain OR cortex OR cortic*). This search returned a total of 154 papers. These terms were used to search the titles and abstracts of articles for their relevance to the research question.

Additionally, a PsycINFO search was conducted using the same search terms which returned 159 papers. This list was then checked for duplicates from the PubMed search which were removed from the list (55 articles) and some were found in reference lists of other papers. A total of 273 abstracts were screened for relevancy. Finally, reference lists of the included studies were searched for studies that might have been missed with the PubMed and PsycINFO search. Studies were screened by 2 reviewers independently.

**Fig. 1:**
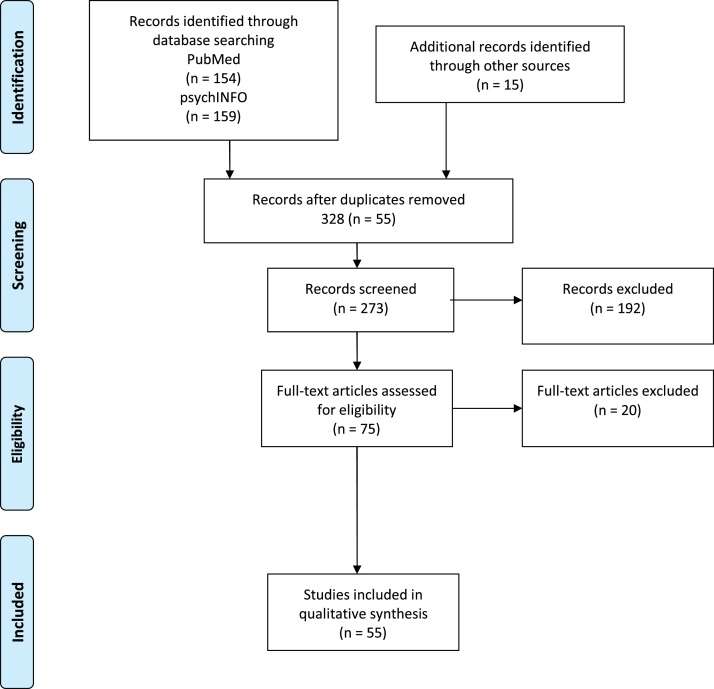
PRISMA diagram detailing review process.

Prospective studies required the use of MRS on both a clinical and a healthy cohort. Studies that did not include a group of individuals with diagnosed schizophrenia, but only high-risk groups, were excluded here. Imaging procedures were required to include an MRS voxel within the frontal lobe of the brain. Patients were designated as either chronic, or first episode patients based on the classification assigned to them by the authors of the original study. Full texts were reviewed for metabolic differences between clinical and control groups, as well as correlations between frontal metabolite concentrations of GABA, Glu, Gln or Glx and cognitive performance. An overview of the review process can be found in Fig. 1 below. Following their inclusion in the review, a study was evaluated using a modified version of the Newcastle-Ottawa Scale (Wells et al., 2000; for details see Appendix A). This evaluation gave the study a mark for quality (out of 6) that attributed to desirable methodological markers. A higher score gave the study a higher degree of relevance and reliability for the factors outlined in this systematic review.

Information about the study design, voxel size and location, participant information (sample size, patient category, medication history), MR field strength and imaging sequence, metabolic measurements, and cognitive measures and/or symptom severity measures were recorded from the studies.

## 3 Results

### 3.1 Study characteristics

From the search described in the methods, 154 papers were acquired through the PubMed database and 159 were acquired from PsychINFO. Following this, 55 papers were removed as they were duplicates found in both database searches. As a result, 258 abstracts were screened to determine their relevance for the research question of this systematic review, of which 182 were subsequently excluded, leaving 76 papers to be examined fully. After examination, a subsequent 20 studies were excluded for a variety of reasons rendering them ineffectual in the current systematic analysis. This left a total of 55 papers included in the current analysis.

36 of the included studies investigated a chronic patient population, and 19 studies involved FEP patients. 28 papers were included in the secondary analysis on cognitive control measures (19 chronic; 9 FES). 4 papers used a combined population that compared metabolite levels of both classifications of patients. A list of studies included in the metabolite comparisons, and further study details can be found in Table 1, Table 2, Table 3, Table 4 (chronic patients) and Table 5, Table 6, Table 7, Table 8 (FEP) below. Studies that reported correlations between frontal GABA, Glu, Gln or Glx metabolite concentrations in SZ patients and cognitive functions or symptom severity are reported in Table 9, Table 10, Table 11 (chronic patients) and Table 12, Table 13 (FEP). Additionally, a summary of the step-by-step details of database search, study selection and exclusion can be seen in the PRISMA diagram in Fig. 1. To assess the quality of studies selected for inclusion, modifications were made to the Newcastle-Ottawa Scale (NOS; Lo et al., 2014) to optimise relevance to the appropriate research methods and participant samples. Details on the factors to which quality was evaluated, and how each included study was rated is presented in the Appendix A.

**Table 1:** Chronic patients - group differences in GABA.

Authors	Study design	Voxel size and location	Sample (medication)	Field strength, spectroscopy scanning sequence	Neurome-tabolites	modified Newcastle-Ottawa score	Results
*Studies showing **decreased** GABA concentrations in chronic patients*							
Marsman et al. (2014)	chronic SZ patients vs. HC	2 × 2 × 2 cm^3^ voxel in medial frontal cortex	16 medicated chronic SZ patients; 23 HC	7 T; MEGA-sLASER	GABA/Cr	6	↓ GABA/Cr in patients compared to HC
Marenco et al. (2016)	treated and untreated chronic SZ patients vs. HC	2 × 2 × 4.5 cm^3^ voxel in medial frontal cortex	83 treated patients; 25 untreated patients; 31 unaffected siblings; 184 HC	3 T; J-edited	GABA/Cr	6	↓ GABA/Cr levels (but not GABA/Water) in treated patients; no difference between untreated patients and HC
					GABA/Water		
Rowland et al. (2013)	chronic SZ patients vs. HC, age of patients considered	3.5 × 3.5 × 3.5 cm^3^ voxel in medial frontal area	21 chronic SZ patients (various APs); 20 HC	3 T; PRESS sequence	GABA	6	Trend towards ↓ GABA in older patients
Rowland et al. (2016a)	older and younger chronic SZ patients vs. age-matched HC groups	4 × 3 × 2 cm^3^ voxel in bilateral medial frontal cortex	29 younger SZ patients (mean age 25.7 ± 4.3 years); 40 younger HC (mean age: 25.3 ± 4.6 y); 31 older SZ patients (mean age: 48.3 ± 5.8 y); 37 older HC (mean age: 51.0 ± 6.0 y); AP medication in majority of patients	3 T; MEGA-PRESS sequence	GABA	5	↓ GABA levels in older SZ patients compared to their age-matched control group; no difference between younger patients and their controls

*Studies showing **increased** GABA concentrations in chronic patients*							
Kegeles et al. (2012)	medicated and unmedicated chronic SZ patients vs. HC	2.5 × 3 × 2.5 cm^3^ voxels in medial frontal and DLPFC areas	16 unmedicated patients; 16 medicated patients; 22 HC	3 T; J-edited spin-echo difference	GABA	5	30% ↑ in GABA in **unmedicated** patients in medial frontal areas compared to HC. No difference in medicated patients. No group differences in DLPFC

*Studies showing **no difference** in GABA concentrations between patients and controls*							
Hjelmervik et al. (2020)	chronic SZ patients with varying degrees of auditory hallucinations vs. HC	4 × 4 × 2.5 cm^3^ voxel in medial frontal cortex	77 medicated chronic patients; 77 HC	3 T; MEGA-PRESS sequence	GABA	6	No difference in GABA levels between groups
Shukla et al. (2019)	chronic SZ patients vs. HC	4 × 3 × 2 cm^3^ voxel in medial frontal cortex	58 chronic patients; 61 HC	3 T; STEAM sequence	GABA	6	No difference in GABA levels between groups
Tayoshi et al. (2010)	chronic patients vs. HC	3 × 3 × 3 cm^3^ voxel in medial frontal cortex	38 chronic patients (various AP); 29 HC	3 T; MEGA-PRESS sequence	GABA	6	No difference in GABA levels between groups
Kegeles et al. (2012)	medicated and unmedicated chronic SZ patients vs. HC	2.5 × 3 × 2.5 cm^3^ voxels in medial frontal and DLPFC areas	16 unmedicated patients; 16 medicated patients; 22 HC	3 T; J-edited spin-echo difference	GABA	5	No difference in **medicated** patients. No group differences in DLPFC
Rowland et al. (2016b)	patients with SZ or schizoaffective disorder vs. HC	medial frontal cortex	45 patients with schizophrenia or schizoaffective disorder; 53 HC	3 T; sequence optimized for glutamatergic measures and GABA	GABA	4	No difference in GABA levels between groups

MRS studies reporting GABA concentrations in frontal brain areas of chronic schizophrenia (SZ) patients, ordered by direction of effect (decrease, increase, no difference), field strength of the MR, study quality according to their score on the modified Newcastle-Ottawa scale (0–6; see Appendix A (Kumar et al., 2020)) and sample size. HC: healthy control participants; AP: antipsychotics; DLPFC: dorsolateral prefrontal cortex.

**Table 2:** Chronic patients - group differences in glutamate (Glu).

Authors	Study design	Voxel size and location	Sample (medication)	Field strength, spectroscopy scanning sequence	Neurometabolites	modified Newcastle-Ottawa score	Results
*Studies showing **decreased** Glu concentrations in chronic patients*							
Kumar et al. (2020)	chronic SZ patients vs. HC	2.0 × 1.8 × 2.5 cm^3^ voxel in medial frontal cortex	28 chronic SZ patients; 45 HC	7 T; STEAM sequence	Glu	6	↓ Glu levels in patients
Théberge et al. (2003)	chronic SZ patients vs. HC	1.5 × 1.5 × 1.5 cm^3^ voxel in left medial frontal cortex	21 chronic patients (various AP); 21 HC	4 T	Glu	6	↓ Glu levels in patients
Shukla et al. (2019)	chronic SZ patients vs. HC	4 × 3 × 2 cm^3^ voxel in medial frontal cortex	58 chronic patients; 61 HC	3 T; STEAM sequence	Glu	6	↓ Glu levels in patients (covarying with age)
Chiappelli et al. (2018)	chronic patients vs. HC	4 × 3 × 2 cm^3^ voxel in medial frontal cortex	56 medicated chronic patients; 58 HC	3 T; STEAM sequence	Glu	6	↓ Glu levels in patients
Chiappelli et al. (2015)	chronic patients vs. HC; metabolite correlations with age	Forceps minor area of left hemisphere	38 chronic patients (age rage: 20−58); 36 HC (age range: 20−61)	3 T; Single Voxel PRESS	Glu	6	↓ Glu with age; greater reductions in patients
Gallinat et al. (2016)	chronic SZ patients vs. HC	2 × 3 × 2 cm^3^ voxel in medial frontal cortex	29 medicated chronic patients; 29 HC	3 T; PRESS sequence	Glu	6	↓ Glu in medial frontal areas; Glu weakly correlated with illness duration
Rowland et al. (2016b)	patients with SZ or schizoaffective disorder vs. HC	medial frontal cortex	45 patients with schizophrenia or schizoaffective disorder; 53 HC	3 T; sequence optimized for glutamatergic measures and GABA	Glu, ratio: glutamine/glutamate,	4	↓ Glu levels in patients compared to HC; ratio Gln/Glu did not differ between groups

*Studies showing **increased** Glu concentrations in chronic patients*							
Tebartz van Elst et al. (2005)	chronic SZ patients vs. HC	2 × 2 × 2 cm^3^ voxel in left DLPFC	21 chronic patients (various AP); 32 HC	2 T; PRESS sequence	Glu	6	↑ Glu in patients
Rüsch et al. (2008)	chronic SZ patients vs. HC	2 × 2 × 2 cm^3^ voxel in left DLPFC	20 chronic medicated SZ patients; 22 HC	2 T; PRESS sequence	Glu	6	↑ Glu in patients

*Studies showing **no difference** in Glu concentrations between patients and controls*							
Marsman et al. (2014)	chronic SZ patients vs. HC	2 × 2 × 2 cm^3^ voxel in medial frontal cortex	16 medicated chronic SZ patients; 23 HC	7 T; MEGA-sLASER	Glu	6	No difference in Glu levels between groups
Bustillo et al. (2014)	chronic patients vs. HC	2 × 2 × 3 cm^3^ voxel in medial frontal cortex	84 chronic patients; 81 HC	3 T; PRESS sequence	Glu	6	No difference in Glu levels between groups
Kaminski et al. (2020)	medicated and unmedicated chronic SZ patients, n-back task during functional MRS	4 × 1 × 2 cm^3^ voxel in left DLPFC	36 medicated chronic SZ patients; 19 unmedicated chronic SZ patients; 35 HC	3 T; point-resolved spectroscopy	Glu	6	No difference in Glu levels between groups
Shah et al. (2020)	chronic SZ patients with different responses to treatment vs. HC	3 × 3 × 3 cm^3^ voxel in medial frontal cortex	24 ultra-treatment resistant patients; 25 patients responsive to clozapine; 19 responsive to non-clozapine AP; 26 HC	3 T; PRESS sequence	Glu	6	No difference in Glu levels
Shirayama et al. (2010)	chronic SZ patients vs. HC	2.8 × 3.0 × 2.2 cm^3^ voxel in medial frontal cortex	19 chronic SZ patients; 18 HC	3 T; PRESS sequence	Glu	6	No difference in Glu levels between groups
Goldstein et al. (2015)	patients with different degrees of treatment resistance vs. HC	1.5 × 1.5 × 3.5 cm^3^ voxel in medial frontal cortex; 2 × 2 × 2 cm^3^ voxel in DLPFC	15 patients: first-line responders; 16 treatment resistant patients taking clozapine (TRS); 11 treatment resistant patients taking different APs after failed clozapine therapy (UTRS); 16 HC	3 T; PRESS sequence	Glu/Cr	6	No group differences for Glu/Cr at either site
Stanley et al. (1996)	first-episode SZ patients, chronic SZ patients and HC	2 × 2 × 2 cm^3^ voxel in left DLPFC	12 chronic patients; 13 FEP (AP naïve) patients; 24 HC	1.5 T; STEAM sequence	Glu	6	No difference in Glu levels between groups
Girgis et al. (2019)	chronic SZ patients vs. HC	2.5 × 3 × 2.5 cm^3^ voxel in medial frontal cortex	19 chronic SZ patients; 20 HC	3 T; CT-PRESS	Glu	5	No difference in Glu levels between groups

MRS studies reporting glutamate (Glu) concentrations in frontal brain areas of chronic schizophrenia (SZ) patients, ordered by direction of effect (decrease, increase, no difference), field strength of the MR, study quality according to their score on the modified Newcastle-Ottawa scale (0–6; see Appendix A) and sample size. HC: healthy control participants; FEP: first-episode patients; AP: antipsychotics; DLPFC: dorsolateral prefrontal cortex.

**Table 3:** Chronic patients - group differences in glutamine (Gln).

Authors	Study design	Voxel size and location	Sample (medication)	Field strength, spectroscopy scanning sequence	Neurome-tabolites	modified Newcastle-Ottawa score	Results
*Studies showing **decreased** Gln concentrations in chronic patients*							
Kumar et al. (2020)	chronic SZ patients vs. HC	2.0 × 1.8 × 2.5 cm^3^ voxel in medial frontal cortex	28 chronic SZ patients; 45 HC	7 T; STEAM sequence	Gln	6	↓ Gln levels in patients
Théberge et al. (2003)	chronic SZ patients vs. HC	1.5 × 1.5 × 1.5 cm^3^ voxel in left medial frontal cortex	21 chronic patients (various AP); 21 HC	4 T	Gln	6	↓ Gln levels in patients

*Studies showing **increased** Gln concentrations in chronic patients*							
Bustillo et al. (2014)	chronic patients vs. HC	2 × 2 × 3 cm^3^ voxel in medial frontal cortex	84 chronic patients; 81 HC	3 T; PRESS sequence	Gln	6	↑ Gln and ↑ Gln/Glu ratio in patients; Gln increased with age
					Gln/Glu		
Tebartz van Elst et al. (2005)	chronic SZ patients vs. HC	2 × 2 × 2 cm^3^ voxel in left DLPFC	21 chronic patients (various AP); 32 HC	2 T; PRESS sequence	Gln	6	↑ Gln in patients
Rüsch et al. (2008)	chronic SZ patients vs. HC	2 × 2 × 2 cm^3^ voxel in left DLPFC	20 chronic medicated SZ patients; 22 HC	2 T; PRESS sequence	Gln	6	↑ Gln in patients
Stanley et al. (1996)	first-episode SZ patients, chronic SZ patients and HC	2 × 2 × 2 cm^3^ voxel in left DLPFC	12 chronic patients; 13 FEP (AP naïve) patients; 24 HC	1.5 T; STEAM sequence	Gln	6	↑ Gln in medicated chronic patients compared to HC

*Studies showing **no difference** in Gln concentrations between patients and controls*							
Shirayama et al. (2010)	chronic SZ patients vs. HC	2.8 × 3.0 × 2.2 cm^3^ voxel in medial frontal cortex	19 chronic SZ patients; 18 HC	3 T; PRESS sequence	Gln	6	No overall group difference, but correlation between Glu/Gln ratio and illness duration
					Gln/Glu		
Rowland et al. (2016b)	patients with SZ or schizoaffective disorder vs. HC	medial frontal cortex	45 patients with schizophrenia or schizoaffective disorder; 53 HC	3 T; sequence optimized for glutamatergic measures and GABA	Gln/Glu ratio	4	Gln/Glu ratio did not differ between groups

MRS studies reporting glutamine (Gln) concentrations in frontal brain areas of chronic schizophrenia (SZ) patients, ordered by direction of effect (decrease, increase, no difference), field strength of the MR, study quality according to their score on the modified Newcastle-Ottawa scale (0–6; see Appendix A) and sample size. HC: healthy control participants; FEP: first-episode patients; AP: antipsychotics; DLPFC: dorsolateral prefrontal cortex.

**Table 4:** Chronic patients - group differences in glutamate + glutamine (Glx).

Authors	Study design	Voxel size and location	Sample (medication)	Field strength, spectroscopy scanning sequence	Neurome-tabolites	modified Newcastle-Ottawa score	Results
*Studies showing **decreased** Glx concentrations in chronic patients*							
Bustillo et al. (2017)	chronic SZ patients vs. HC; comparisons across age ranges	2.3 cm^3^ voxel volume in medial frontal areas	104 chronic SZ patients; 96 HC	3 T PRESS sequence	Glx	6	↓ Glx in patients regardless of age
Hjelmervik et al. (2020)	chronic SZ patients with varying degrees of auditory hallucinations vs. HC	4 × 4 × 2.5 cm^3^ voxel in medial frontal cortex	77 medicated chronic patients; 77 HC	3 T; MEGA-PRESS sequence	Glx	6	↓ Glx only in patients with more auditory hallucinations compared to HC
Ćurčić-Blake et al. (2017)	chronic SZ patients vs. HC	8 × 8 × 8 cm^3^ voxel in left frontal lobe	67 chronic patients; 30 HC	3 T; PRESS sequence	Glx	6	↓ Glx in patients compared to HC
Liemburg et al. (2016)	chronic patients, first episode patients, high risk individuals and HC	2 × 2 × 2 cm^3^ voxel in medial frontal cortex	60 chronic patients; 31 recent onset patients; 16 Ultra High Risk individuals; 36 controls	3 T; PRESS sequence	Glx	6	↓ Glx in chronic patients compared to HC; negative correlation with illness duration
Cadena et al. (2018)	chronic SZ patients vs. HC, MRS measured at baseline and after 6 weeks of AP use	2.7 × 2 × 1 cm^3^ voxel in medial frontal cortex	28 SZ patients (off APs for at least 10 days); 25 HC	3 T; PRESS sequence	Glx (relative to Cr)	6	↓ Glx/Cr ratio after AP medication; no baseline difference before AP usage
Natsubori et al. (2014)	chronic SZ, first episode, high genetic risk individuals and HC	2 × 2 × 2 cm^3^ voxel in medial frontal cortex	25 chronic SZ patients; 19 FEP; 24 Ultra High Genetic Risk (UHR);Matched HC for each group	3 T; STEAM sequence	Glx	6	↓ Glx in chronic SZ patients compared to HC; other groups showed reductions to lesser degree.
Hugdahl et al. (2015)	chronic SZ patients vs. HC	4 voxels (2 × 2 × 2 cm^3^); left and right frontopolar locations, left and right temporal cortex	23 chronic SZ patients (majority with AP); 26 HC	3 T; GE single-voxel PRESS sequence	Glx	6	↓ Glx in patients compared to HC in frontal voxels
Rowland et al. (2013)	chronic SZ patients vs. HC, age of patients considered	3.5 × 3.5 × 3.5 cm^3^ voxel in medial frontal area	21 chronic SZ patients (various APs); 20 HC	3 T; PRESS sequence	Glx	6	↓ Glx in patients irrespective of age
Ohrmann et al. (2007)	chronic SZ patients, first-episode patients and HC	3.375 cm^3^ voxel in left DLPFC	20 chronic medicated patients; 15 FEP neuroleptic-naïve patients; 20 HC	1.5 T; single-voxel STEAM sequence	Glx	5	↓ Glx in chronic patients compared to HC and compared to FEP patients; medication had no impact on metabolite levels in chronic patients
Ohrmann et al. (2005)	chronic SZ patients, first episode patients and HC	DLPFC (voxel size not reported)	21 chronic SZ patients; 18 first-episode patients; 21 HC	1.5 T; proton-density weighted fast spin echo sequences	Glx	5	↓ Glx in chronic patients compared to both first-episode patients and HC

*Studies showing **increased**Glx concentrations in chronic patients*							
Chang et al. (2007)	elderly SZ patients with cognitive decline vs. age-matched HC	voxel in frontal brain regions, unknown voxel size	23 elderly chronic SZ patients; 22 HC	4 T; Optimized double spin echo sequence	Glx	5	↑ Glx in patients
Hjelmervik et al. (2020)	chronic SZ patients with varying degrees of auditory hallucinations vs. HC	4 × 4 × 2.5 cm^3^ voxel in medial frontal cortex	77 medicated chronic patients; 77 HC	3 T; MEGA-PRESS sequence	Glx	6	↑ Glx only in patients with fewer auditory hallucinations compared to HC
Kegeles et al. (2012)	medicated and unmedicated chronic SZ patients vs. HC	2.5 × 3 × 2.5 cm^3^ voxels in medial frontal and DLPFC areas	16 unmedicated patients; 16 medicated patients; 22 HC	3 T; J-edited spin-echo difference	Glx	5	30% ↑ in Glx only in **unmedicated** patients in medial frontal areas compared to HC. No difference in medicated patients. No group differences in DLPFC

*Studies showing **no difference** in Glx concentrations between patients and controls*							
Kraguljac et al. (2018)	chronic SZ patients vs. HC; measurements before and after 6 weeks of risperidone usage	2.7 × 2 × 1 cm^3^ voxels in medial frontal cortex and hippocampus	61 chronic patients; 31 HC	3 T; PRESS sequence	Glx	6	No difference in Glx levels before or after AP usage in medial frontal cortex
Chiappelli et al. (2018)	chronic patients vs. HC	4 × 3 × 2 cm^3^ voxel in medial frontal cortex	56 medicated chronic patients; 58 HC	3 T; STEAM sequence	Glx	6	No difference in Glx levels between groups
Reid et al. (2010)	MRS and fMRI measures during a Stroop task in SZ patients and HC	2.7 × 2 × 1 cm^3^ voxel in medial frontal cortex	26 chronic SZ patients; 23 HC	3 T; PRESS sequence	Glx (relative to Cr)	6	No difference in Glx ratio between groups
Shah et al. (2020)	chronic SZ patients with different responses to treatment vs. HC	3 × 3 × 3 cm^3^ voxel in medial frontal cortex	24 ultra-treatment resistant patients; 25 patients responsive to clozapine; 19 responsive to non-clozapine AP; 26 HC	3 T; PRESS sequence	Glx	6	No overall group differences; negative correlation between dACC Glx levels and cortical thickness in DLPFC
Coughlin et al. (2015)	chronic SZ patients vs. HC	3.5 × 3.5 × 3.5 cm^3^ voxels in medial and lateral frontal cortex	25 medicated chronic patients; 17 HC	3 T; PRESS sequence	Glx (relative to Cr)	6	No group difference in either region
Rowland et al. (2009)	chronic SZ patients vs. HC	1.5 × 1.5 × 1.5 cm^3^ voxel in left DLPFC (middle frontal gyrus)	18 chronic patients; 10 HC	3 T; PRESS sequence	Glx	6	No Glx difference between groups
Goldstein et al. (2015)	patients with different degrees of treatment resistance vs. HC	1.5 × 1.5 × 3.5 cm^3^ voxel in medial frontal cortex; 2 × 2 × 2 cm^3^ voxel in DLPFC	15 patients: first-line responders; 16 treatment resistant patients taking clozapine (TRS); 11 treatment resistant patients taking different APs after failed clozapine therapy (UTRS); 16 HC	3 T; PRESS sequence	Glu/Cr	6	No group differences for Glu/Cr at either voxel site; Higher Glx/Cr levels in DLPFC of first-line responders than in UTRS, but no difference between patient groups and HC.
					Glx/Cr		
Kegeles et al. (2012)	medicated and unmedicated chronic SZ patients vs. HC	2.5 × 3 × 2.5 cm^3^ voxels in medial frontal and DLPFC areas	16 unmedicated patients; 16 medicated patients; 22 HC	3 T; J-edited spin-echo difference	Glx	5	No Glx difference between **medicated** patients and HC
Ota et al. (2012)	chronic SZ patients vs HC	1.5 × 2.5 × 2 cm^3^ voxel in frontal white matter regions	22 medicated chronic SZ patients; 27 HC	1.5 T; PRESS sequence	Glx	6	No Glx difference between groups
Szulc et al. (2013)	chronic SZ patients (7 days after neuroleptic cessation and again after 4 weeks neuroleptic treatment) vs. HC	2 × 2 × 2 cm^3^ voxel in left frontal areas	17 treatment responders; 23 non-responders; 25 HC	1.5 T single-voxel PRESS	Glx/Cr	5	No difference between patients and HC; but ↓ Glx in treatment responders compared to non-responders

MRS studies reporting glutamate + glutamine (Glx) concentrations in frontal brain areas of chronic schizophrenia (SZ) patients, ordered by direction of effect (decrease, increase, no difference), field strength of the MR, study quality according to their score on the modified Newcastle-Ottawa scale (0–6; see Appendix A) and sample size. HC: healthy control participants; FEP: first-episode patients; AP: antipsychotics; DLPFC: dorsolateral prefrontal cortex.

**Table 5:** First-episode patients - group differences in GABA.

Authors	Study design	Voxel location and size	Sample	Spectroscopy scanning sequence	Neurometabolites	modified Newcastle-Ottawa score	Results
*Studies showing **decreased** GABA concentrations in FEP*							
Wang et al. (2019)	FEP vs. HC	2 × 3 × 2 cm^3^ voxel in medial frontal areas; 2 × 2.5 × 2 cm^3^ voxel in left DLPFC	81 medicated FEP; 91 HC	7 T; STEAM sequence	GABA	6	↓ GABA levels in medial frontal areas in FEP; no differences in DLPFC
Bojesen et al. (2020)	FEP vs. HC; longitudinal study to measure treatment response	2 × 2 × 2 cm^3^ voxel in medial frontal areas	39 FEP; 36 HC	3 T; PRESS	GABA	6	Only treatment non-responders: ↓ GABA in medial frontal areas
Wang et al. (2016)	FEP vs. HC	3 × 3 × 3 cm^3^ voxel in medial frontal areas	16 AP naïve FEP, 23 HC	3 T; MEGA-PRESS sequence	GABA	6	↓ GABA levels in unmedicated FEP

*Studies showing **increased** GABA concentrations in FEP*							
De la Fuente-Sandoval et al. (2017)	antipsychotic-naïve patients vs. HC	3 × 2.5 × 2.5 cm^3^ voxel in medial frontal areas	28 FEP (AP naïve at baseline, then treated with risperidone for 4 weeks); 18 HC	3 T; J-edited spin echo	GABA	6	↑ GABA in unmedicated FEP (at baseline); after 4 weeks of treatment: no difference in GABA compared to HC
Cen et al. (2020)	FEP vs. HC	3 × 3 × 3 cm^3^ voxel in ventromedial prefrontal areas	23 FEP (AP naïve); 26 HC	3 T; MEGA-PRESS	GABA+	6	↑ GABA + in patients

*Studies showing **no difference** in GABA concentrations between patients and controls*							
Reid et al. (2019)	FEP vs. HC	2.7 × 2.0 × 1.0 cm^3^ voxel in medial frontal areas	21 FEP; 21 HC	7 T; STEAM	GABA	6	No difference in GABA levels between groups
De la Fuente-Sandoval et al. (2017)	antipsychotic-naïve patients vs. HC	3 × 2.5 × 2.5 cm^3^ voxel in medial frontal areas	28 FEP (AP naïve at baseline, then treated with risperidone for 4 weeks);18 HC	3 T; J-edited spin echo	GABA	6	After 4 weeks of AP treatment: no difference in GABA compared to HC
Goto et al. (2010)	FEP vs. HC	3 × 3 × 3 cm^3^ voxel in frontal lobe	18 medicated FEP; 18 HC	3 T; MEGA-PRESS	GABA	6	No difference in GABA levels between groups

MRS studies reporting GABA concentrations in frontal brain areas of first-episode patients (FEP), ordered by direction of effect (decrease, increase, no difference), field strength of the MR, study quality according to their score on the modified Newcastle-Ottawa scale (0–6; see Appendix A) and sample size. HC: healthy control participants; AP: antipsychotics; DLPFC: dorsolateral prefrontal cortex.

**Table 6:** First-episode patients - group differences in glutamate (Glu).

Authors	Study design	Voxel location and size	Sample	Spectroscopy scanning sequence	Neurometabolites	modified Newcastle-Ottawa score	Results
*Studies showing **decreased** Glu concentrations in FEP*							
Wang et al. (2019)	FEP vs. HC	2 × 3 × 2 cm^3^ voxel in medial frontal areas; 2 × 2.5 × 2 cm^3^ voxel in left DLPFC	81 medicated FEP;91 HC	7 T; STEAM sequence	Glu	6	↓ Glu levels in medial frontal areas in FEP; no differences in DLPFC
Reid et al. (2019)	FEP vs. HC	2.7 × 2.0 × 1.0 cm^3^ voxel in medial frontal areas	21 FEP; 21 HC	7 T; STEAM	Glu	6	↓ Glu levels in FEP
Bojesen et al. (2020)	FEP vs. HC; longitudinal study to measure treatment response	2 × 2 × 2 cm^3^ voxel in medial frontal areas	39 FEP; 36 HC	3 T; PRESS	Glu/Cr	6	↓ Glu/Cr in FEP patients compared to HC

*Studies showing **increased** Glu concentrations in FEP*							
Olbrich et al. (2008)	FEP vs. HC	2 × 2 × 2 cm^3^ voxel in left DLPFC	9 medicated FEP; 32 HC	2 T; PRESS sequence	Glu	6	↑ Glu levels in patients compared to HC

*Studies showing **no difference** in Glu concentrations between patients and controls*							
Dempster et al. (2020)	FEP vs. HC, measuring treatment response	2 × 2 × 2 cm^3^ voxel in medial frontal areas	36 FEP (minimal treatment); 27 HC	7 T; Semi-LASER	Glu	6	No difference in Glu
Théberge et al. (2002)	medication-naïve FEP vs. HC	1.5 × 1.5 × 1.5 cm^3^ voxel in left medial frontal areas	21 FEP (medication naïve); 21 HC	4 T; stimulated echo acquisition	Glu	6	No difference in Glu
Aoyama et al. (2011)	FEP vs. HC	1.5 × 1.5 × 1.5 cm^3^ voxel in medial frontal areas	17 FEP; 17 HC	4 T; STEAM	Glu	6	No difference in Glu; no difference between medication-naïve and previously treated patients
Bustillo et al. (2010)	FEP measured before and after AP use vs. HC	2 × 2 × 2 cm^3^ voxel in medial frontal areas	14 FEP (minimal AP); 10 HC	4 T; STEAM	Glu	5	No difference in Glu
Li et al. (2020)	Longitudinal design: drug-naïve FEP scanned at baseline and after 8 weeks of risperidone treatment vs. HC	medial frontal (pregenual anterior cingulate cortex)	35 drug-naïve first-episode patients; 40 HC	3 T; PRESS sequence	Glu Glu/Cr + PCr (total creatine)	6	No difference in Glu and Glu/Cr + PCr in ACC between patients and controls at baseline
Jauhar et al. (2018)	FEP vs. HC	2 × 2 × 2 cm^3^ voxel in medial frontal areas	28 FEP; 20 HC	3 T; PRESS	Glu	6	No difference in Glu
Stanley et al. (1996)	FEP vs. chronic patients vs. HC	2 × 2 × 2 cm^3^ voxel in left DLPFC	13 FEP (AP naïve); 12 chronic patients; 24 HC	1.5 T; STEAM	Glu	6	No difference in Glu

MRS studies reporting glutamate (Glu) concentrations in frontal brain areas of first-episode patients (FEP), ordered by direction of effect (decrease, increase, no difference), field strength of the MR, study quality according to their score on the modified Newcastle-Ottawa scale (0–6; see Appendix A) and sample size. HC: healthy control participants; AP: antipsychotics, DLPFC: dorsolateral prefrontal cortex.

**Table 7:** First-episode patients - group differences in glutamate (Gln).

Authors	Study design	Voxel location and size	Sample	Spectroscopy scanning sequence	Neurometabolites	modified Newcastle-Ottawa score	Results
*Studies showing **increased** Gln concentrations in FEP*							
Théberge et al. (2002)	medication-naïve FEP vs. HC	1.5 × 1.5 × 1.5 cm^3^ voxel in left medial frontal areas	21 FEP (medication naïve); 21 HC	4 T; stimulated echo acquisition	Gln	6	↑ Gln in patients
Bustillo et al. (2010)	FEP measured before and after AP use vs. HC	2 × 2 × 2 cm^3^ voxel in medial frontal areas	14 FEP (minimal AP); 10 HC	4 T; STEAM	Gln	5	↑ Gln/Glu ratio in patients before treatment; no difference for Glu or Gln
					Gln/Glu		

*Studies showing **no difference** in Gln concentrations between patients and controls*							
Wang et al. (2019)	FEP vs. HC	2 × 3 × 2 cm^3^ voxel in medial frontal areas; 2 × 2.5 × 2 cm^3^ voxel in left DLPFC	81 medicated FEP; 91 HC	7 T; STEAM sequence	Gln	6	No difference in Gln
Reid et al. (2019)	FEP vs. HC	2.7 × 2.0 × 1.0 cm^3^ voxel in medial frontal areas	21 FEP; 21 HC	7 T;STEAM	Gln	6	No difference in Gln
Aoyama et al. (2011)	FEP vs. HC	1.5 × 1.5 × 1.5 cm^3^ voxel in medial frontal areas	17 FEP; 17 HC	4 T; STEAM	Gln	6	No difference in Gln
Bustillo et al. (2010)	FEP measured before and after AP use vs. HC	2 × 2 × 2 cm^3^ voxel in medial frontal areas	14 FEP (minimal AP); 10 HC	4 T; STEAM	Gln	5	No difference in Gln
					Gln/Glu		
Olbrich et al. (2008)	FEP vs. HC	2 × 2 × 2 cm^3^ voxel in left DLPFC	9 medicated FEP; 32 HC	2 T; PRESS sequence	Gln	6	No difference in Gln
Stanley et al. (1996)	FEP vs. chronic patients vs. HC	2 × 2 × 2 cm^3^ voxel in left DLPFC	13 FEP (AP naïve); 12 chronic patients; 24 HC	1.5 T; STEAM	Gln	6	No difference in Gln

MRS studies reporting glutamine (Gln) concentrations in frontal brain areas of first-episode patients (FEP), ordered by direction of effect (decrease, increase, no difference), field strength of the MR, study quality according to their score on the modified Newcastle-Ottawa scale (0–6; see Appendix A) and sample size. HC: healthy control participants; AP: antipsychotics, DLPFC: dorsolateral prefrontal cortex.

**Table 8:** First-episode patients - group differences in glutamate + glutamine (Glx).

Authors	Study design	Voxel location and size	Sample	Spectroscopy scanning sequence	Neurometabolites	modified Newcastle-Ottawa score	Results
*Studies showing **increased** Glx concentrations in FEP*							
Bartolomeo et al. (2019)	individuals with early phase psychosis (EPP within 5 years of 1 st onset) vs. HC	2 × 2 × 2 cm^3^ voxel in medial frontal cortex	34 EPP; 19 HC	3 T; single voxel PRESS sequence	Glx	6	↑ Glx in EPP patients

*Studies showing **decreased** Glx concentrations in FEP*							
Natsubori et al. (2014)	FEP, chronic SZ patients, high genetic risk individuals and HC	2 × 2 × 2 cm^3^ voxel in medial frontal cortex	19 FEP; 25 chronic SZ patients; 24 Ultra High Genetic Risk; matched HC for each group	3 T STEAM sequence	Glx	6	↓ Glx levels in FEP (but more than chronic patients)
Wang et al. (2016)	FEP vs. HC	3 × 3 × 3 cm^3^ voxel in medial frontal areas	16 AP naïve FEP, 23 HC	3 T; MEGA-PRESS sequence	Glx	6	↓ Glx in FEP

*Studies showing **no difference** in Glx concentrations between patients and controls*							
Liemburg et al (2016)	FEP, chronic patients, ultra-high risk individuals and HC	2 × 2 × 2 cm^3^ voxel in medial frontal cortex	31 FEP;60 chronic patients; 16 UHR; 36 HC	3 T PRESS sequence	Glx	6	No difference in Glx levels
Cen et al. (2020)	FEP vs. HC	3 × 3 × 3 cm^3^ voxel in ventromedial prefrontal areas	23 FEP (AP naïve); 26 HC	3 T; MEGA-PRESS	Glx	6	No difference in Glx levels
Goto et al. (2012)	FEP vs. HC	3 × 3 × 3 cm^3^ voxel in frontal lobe	16 FEP (AP naïve); 18HC	3 T; MEGA-PRESS	Glx/Cr	6	No difference in Glx/Cr ratio
Galinska et al. (2009)	FEP vs. HC, variations in duration of untreated illness	2 × 2 × 2 cm^3^ voxel in left frontal areas	30 FEP (median duration of untreated illness: 10 weeks); 19 HC	1.5 T; PRESS	Glx/Cr	6	No differences in Glx/Cr ratio; no difference between patients with long or short duration of untreated illness
Stanley et al. (1996)	FEP vs. chronic patients vs. HC	2 × 2 × 2 cm^3^ voxel in left DLPFC	13 FEP (AP naïve); 12 chronic patients; 24 HC	1.5 T; STEAM	Glx	6	No difference in Glx levels
Ohrmann et al. (2005)	FEP, chronic patients and HC	DLPFC (voxel size not reported)	18 FEP, 21 chronic patients, 21 HC	1.5 T proton-density weighted fast spin echo sequences	Glx	5	No difference in Glx between FEP and HC
Ohrmann et al. (2007)	FEP, chronic SZ patients and HC	3.4 × 3.4 × 3.4 cm^3^ voxel in left DLPFC	20 chronic medicated patients; 15 FEP neuroleptic-naive patients; 20 HC	1.5 T; single-voxel STEAM sequence	Glx	5	No Glx difference between first-episode patients and HC; but higher Glx levels than chronic patients (p < 0.05)

MRS studies reporting glutamate + glutamine (Glx) concentrations in frontal brain areas of first-episode patients (FEP), ordered by direction of effect (decrease, increase, no difference), field strength of the MR, study quality according to their score on the modified Newcastle-Ottawa scale (0–6; see Appendix A) and sample size. HC: healthy control participants; AP: antipsychotics; DLPFC: dorsolateral prefrontal cortex.

**Table 9:** Chronic patients: Correlations between GABA and cognitive functions or symptom severity.

Authors	Study design	Voxel size and location	Sample and medication	Field strength; spectroscopy scanning sequence	Neurome-tabolites	modified Newcastle-Ottawa score	Assessment tools	Investigated functions/ symptoms	Results
Marsman et al. (2014)	chronic SZ patients vs. HC; correlations with IQ scales and symptom severity	2 × 2 × 2 cm^3^ voxel in medial frontal cortex	16 medicated chronic SZ patients; 23 HC	7 T; MEGA-sLASER	GABA/Cr	6	PANSS; Wechsler Adult Intelligence Scale (WAIS - III)	intelligence, incl. separate subscales; positive and negative symptom severity	*negative correlations:* lower GABA/Cr ratio associated with higher **IQ**, specifically with performance IQ, **WM**, verbal IQ, **perceptual reasoning** and **verbal comprehension**; no sign. association between GABA/CR and symptom severity
Rowland et al. (2013)	chronic SZ patients vs. HC; correlations with attention and WM measures	3.5 × 3.5 × 3.5 cm^3^ voxel in medial frontal area	21 chronic SZ patients (various APs), 20 HC	3 T; PRESS sequence	GABA	6	coding test digit span	attention; working memory	*positive correlation:* higher frontal GABA levels associated with better **attentional performance** (coding test); no sign correlation between WM performance and GABA levels
Xiang et al. (2019)	chronic SZ patients vs HC; correlations with positive and negative symptom scores	3.5 × 2.5 × 3.0 cm^3^ voxel in left DLPFC	20 chronic medicated SZ patients; 26 HC	3 T; MEGA-PRESS sequence	GABA+	6	PANSS	positive and negative symptom severity	*positive correlation:* GABA + level correlated with **PANSS total score**;
Rowland et al. (2016a)	older and younger SZ patients and HC; GABA levels correlated with working memory, processing speed, positive/negative symptom severity	4 × 3 × 2 cm^3^ voxel in bilateral medial frontal cortex	29 younger SZ patients (mean age 25.7 ± 4.3 years); 40 younger HC (mean age: 25.3 ± 4.6 years); 31 older SZ patients (age: 48.3 ± 5.8 years); 37 older HC (mean age: 51.0 ± 6.0 years); majority of patients medicated	3 T; MEGA-PRESS sequence	GABA	5	BPRS	positive and negative symptoms; WM; processing speed	*positive correlation:* GABA levels were predicted by age (declining with age) in SZ group, but not in HC; higher GABA level predicted better **WM**, even when controlling for age;
							Brief Negative Symptom Scale (BNSS); digit sequencing test (WM), digit symbol coding test (processing speed)		
									No sign. relationship between GABA and positive or negative symptom severity (BPRS, BNSS scores) or processing speed.
Kegeles et al. (2012)	dedicated and unmedicated chronic SZ patients vs. HC; correlations with WM	2.5 × 3 × 2.5 cm^3^ voxels in medial frontal and DLPFC areas	16 unmedicated patients; 16 medicated patients; 22 HC	3 T; J-edited spin-echo difference	GABA	5	n-back task	WM	no sign. correlations between either GABA and WM performance or symptom severity
							PANSS		
Rowland et al. (2016b)	mix of patients with early and chronic schizophrenia or schizoaffective disorder and HC; correlations with WM, processing speed and neural correlates of prediction errors	medial frontal cortex	45 chronic, FEP and schizoaffective disorder patients; 53 HC	3 T; sequence optimized for glutamatergic measures and GABA	GABA	4	EEG recordings; BPRS; Digit Sequencing Task (DST) to measure WM; digit symbol coding subtest of WAIS III (processing speed)	modulations in mismatch negativity (MMN); verbal WM, processing speed	*positive correlations:* larger **MMN amplitudes** associated with higher GABA levels in patients, but not in control group; Higher GABA levels associated with better verbal **WM** performance and higher **processing speed** in patients, but not in controls
									No correlation with negative or total BPRS scores.

MRS studies reporting correlations between GABA concentrations in frontal brain areas of chronic schizophrenia (SZ) patients and cognitive functions or severity of other symptoms; BPRS: Brief Psychiatric Rating Scale; PANSS: Positive and Negative Syndrome Scale; WM: working memory; AP: antipsychotics; DLPFC: dorsolateral prefrontal cortex; HC: healthy control participants.

**Table 10:** Chronic patients: Correlations between glutamate (Glu) or glutamine (Gln) and cognitive functions or symptom severity.

Authors	Study design	Voxel size and location	Sample and medication	Field strength; spectroscopy scanning sequence	Neurome-tabolites	modified Newcastle-Ottawa score	Assessment tools	Investigated functions/ symptoms	Results
Marsman et al. (2014)	chronic SZ patients vs. HC; correlations with IQ scales and symptom severity	2 × 2 × 2 cm^3^ voxel in medial frontal cortex	16 medicated chronic SZ patients; 23 HC	7 T; MEGA-sLASER	Glu	6	PANSS; Wechsler Adult Intelligence Scale (WAIS - III)	intelligence, incl. separate subscales; positive and negative symptom severity	No sign. association between Glu and symptom severity
Kaminski et al. (2020)	chronic medicated and unmedicated patients and HC; fMRI during WM task	4 × 1 × 2 cm^3^ voxel in left DLPFC	36 medicated patients; 19 unmedicated patients; 35 HC	3 T; point-resolved spectroscopy	Glu	6	PANSS; fMRI during n-back task	BOLD response in DLPFC in WM task; positive and negative symptom severity	*positive correlation:* between **WM-dependent BOLD activity** in DLPFC and Glu levels in unmedicated patients (but not medicated patients).
									*Negative correlation:* lower Glu levels associated with more **positive symptoms** in medicated, but not unmedicated patients.
Shirayama et al. (2010)	chronic patients and HC; correlations with symptom severity and various cognitive functions	2.8 × 3.0 × 2.2 cm^3^, medial frontal cortex	19 chronic SZ patients; 18 HC	3 T; PRESS sequence	Glu	6	BPRS; Scale for the Assessment of Negative Symptoms (SANS); verbal fluency test;Wisconsin card sorting test (WCST); trail-making test; digit span distraction test (DSDT); Stroop task; Iowa gambling task	positive and negative symptom severity; verbal fluency; set shifting; selective attention; response inhibition; learning from feedback	Gln/Glu ratio correlated with **set shifting performance** (pos. correlation with perseveration errors; neg. correlation with completed WCST categories) and **selective attention** (DSDT); no correlations between neurometabolites and symptom severity and other neuropsychological measures;
					Gln				
					Gln/Glu				
									NOTE: these correlations were calculated across both patient and HC groups
Bustillo et al. (2014)	chronic patients vs. HC; correlations with general cognitive functions and symptom symptom severity	2 × 2 × 3 cm^3^ voxel in medial frontal cortex	84 chronic patients; 81 HC	3 T; PRESS sequence	Glu	6	PANSS; Measurement and Treatment Research to Improve Cognition in Schizophrenia (MATRICS)	positive and negative symptoms severity; general cognitive assessment	no correlation with negative symptoms; no correlation between neurometabolites and general cognitive functions
					Gln				
					Gln/Glu				*positive correlation:* between Gln and **positive symptom** severity
Chiappelli et al. (2015)	chronic patients vs. HC; metabolite correlations with symptom severity, processing speed, WM	Forceps minor area of left hemisphere	38 chronic patients; 36 HC	3 T; Single Voxel PRESS	Glu	6	BPRS; Digit-symbol coding task (processing speed); digit sequencing task (working memory)	positive and negative symptom severity; processing speed; WM	No correlations between Glu levels and symptom severity, processing speed or WM
Rowland et al. (2016b)	mix of patients with early and chronic schizophrenia or schizoaffective disorder and HC; correlations with WM, processing speed and neural correlates of prediction errors	medial frontal cortex	45 chronic, FEP and schizoaffective disorder patients; 53 HC	3 T; sequence optimized for glutamatergic measures and GABA	Glu Gln/Glu ratio	4	EEG recordings; BPRS; Digit Sequencing Task (DST) to measure WM; digit symbol coding subtest of WAIS III (processing speed)	modulations in mismatch negativity (MMN); verbal WM, processing speed	*positive correlation:* larger **MMN amplitudes** associated with higher Glu levels, and with lower glutamine/glutamate ratio in patients, but not in control group;
									No sign. correlations with negative or total BPRS scores.
Tebartz van Elst et al. (2005)	chronic patients vs. HC; correlation with psychosocial functioning	2 × 2 × 2 cm^3^ voxel in left DLPFC	21 chronic patients (various AP); 32 HC	2 T; PRESS sequence	Glu	6	Global assessment of function scale	psychosocial functioning	*negative correlation:* higher lateral frontal Glu concentrations associated with worse overall **psychosocial functioning** over last 2 years; No correlations reported for Gln
					Gln				
Rüsch et al. (2008)	chronic SZ patients vs. HC; correlations with different WCST measures	2 × 2 × 2 cm^3^ voxel in left DLPFC	20 chronic medicated SZ patients; 22 HC	2 T	Glu	6	Wisconsin Card Sorting Test (WCST)	executive functions	No sign. correlations in DLPFC in SZ patients
				PRESS sequence	Gln				

MRS studies reporting correlations between glutamate (Glu) or glutamine (Gln) concentrations in frontal brain areas of chronic schizophrenia (SZ) patients and cognitive functions or severity of other symptoms; BPRS: Brief Psychiatric Rating Scale; PANSS: Positive and Negative Syndrome Scale; RBANS: Repeatable Battery for the Assessment of Neuropsychological Status; WM: working memory; DLPFC: dorsolateral prefrontal cortex; HC: healthy control participants.

**Table 11:** Chronic patients: Correlations between glutamate + glutamine (Glx) and cognitive functions or symptom severity.

Authors	Study design	Voxel size and location	Sample and medication	Field strength; spectroscopy scanning sequence	Neurome-tabolites	modified Newcastle-Ottawa score	Assessment tools	Investigated functions/ symptoms	Results
Bustillo et al. (2011)	older and younger chronic schizophrenia patients and corresponding HC	different ROIs within 1 acquired slice; nominal voxel size 1 × 1 × 1 cm^3^	30 patients (12 young, 18 older); 28 HC (10 young, 18 older)	4 T; PEPSI sequence	Glx	6	large range of different neuropsychological tests	cognitive measures were combined into 3 factors, resulting in general cognitive measure that was correlated with Glx	*positive correlation:* higher Glx concentration associated with higher **cognitive performance** factor score
Liemburg et al. (2016)	chronic patients, first episode patients, high risk individuals and HC; correlations with symptom severity	2 × 2 × 2 cm^3^ voxel in medial frontal cortex	60 chronic patients; 31 recent onset patients; 16 Ultra High Risk individuals; 36 controls	3 T; PRESS sequence	Glx	6	PANSS	positive and negative symptoms	No sign. correlation between symptom severity and Glx
Reid et al. (2010)	chronic patients and HC; correlations with different cognitive control measures	2.7 × 2 × 1 cm^3^ voxel in medial frontal cortex	26 chronic patients; 23 HC	3 T; PRESS sequence	Glx/Cr	6	Stroop task; RBANS; BPRS	Stroop interference effect, post-conflict adjustment, post-error slowing	*negative correlation:* lower Glx/Cr ratio associated with more **negative symptoms** (BPRS);
									No sign. correlations between Glx ratio and either Stroop interference effect or RBANS total score
Hugdahl et al. (2015)	chronic SZ vs. HC individuals; MRS measures correlated with PANSS scores	4 voxels (2 × 2 × 2 cm^3^); left and right frontopolar locations, left and right temporal cortex	23 patients; patients (majority medicated); 26 HC	3 T; PRESS sequence MRS data scaled to water reference and adjusted for partial volume effects	Glx	6	PANSS and individual subscale scores	positive and negative symptoms; specifically P3 hallucination symptom scores and N2 emotional withdrawal scores	*positive correlation*: frontal Glx associated with **P3 hallucination symptom score** and sum total of **positive symptoms**; no sign. association between N2 scores or sum total of negative symptoms and Glx levels
Rowland et al. (2013)	chronic SZ patients vs. HC; correlations with attention and WM measures	3.5 × 3.5 × 3.5 cm^3^ voxel in medial frontal area	21 chronic SZ patients (various APs), 20 HC	3 T; PRESS sequence	Glx	6	coding test digit span	attention; working memory	No sign correlation between WM performance and Glx levels
Xiang et al. (2019)	chronic SZ patients vs HC; correlations with positive and negative symptom scores	3.5 × 2.5 × 3.0 cm^3^ voxel in left DLPFC	20 chronic medicated SZ patients; 26 HC	3 T; MEGA-PRESS sequence	Glx	6	PANSS	positive and negative symptom severity	*positive correlation:* Glx correlated with **negative symptom severity**
Rowland et al. (2009)	chronic patients vs. HC; correlations with neuropsychological status and symptom severity	1.5 × 1.5 × 1.5 cm^3^ voxel in left DLPFC	18 chronic patients; 10 HC	3 T; PRESS sequence	Glx	6	RBANS; BPRS, SANS	positive and negative symptoms; RBANS score	No sign. correlations with symptom severity or RBANS score
Goldstein et al. (2015)	patients with different degrees of treatment resistance vs. HC	1.5 × 1.5 × 3.5 cm^3^ voxel in medial frontal cortex;2 × 2 × 2 cm^3^ voxel in DLPFC	15 patients: first-line responders; 16 treatment resistant patients taking clozapine (TRS); 11 treatment resistant patients taking different APs after failed clozapine therapy (UTRS); 16 HC	3 T; PRESS sequence	Glx/Cr	6	PANSS	positive and negative symptoms	No sign. correlations with PANSS scores
Kegeles et al. (2012)	dedicated and unmedicated chronic SZ patients vs. HC; correlations with WM	2.5 × 3 × 2.5 cm^3^ voxels in medial frontal and DLPFC areas	16 unmedicated patients; 16 medicated patients; 22 HC	3 T; J-edited spin-echo difference	Glx	5	n-back task	WM	No sign. correlations between Glx and WM performance or symptom severity
							PANSS		
Ohrmann et al. (2008)	chronic patients vs. HC; correlations with attentional functions, memory, reasoning and symptom severity	1.5 × 1.5 × 1.5 cm^3^ voxel in left DLPFC; 1.5 × 1.5 × 1.5 cm^3^ voxel in medial frontal cortex	43 chronic medicated patients, 37 HC	1.5 T; PRESS sequence	Glx	6	WCST; Auditory Verbal Learning Task; Frankfurt Attention Inventory; LPS perceptual reasoning task; PANSS	set shifting, memory, attention, perceptual reasoning; positive and negative symptoms	*positive correlation:* between medial frontal (but not lateral frontal) Glx and **set shifting** improvements; no sign. correlations with memory performance, processing speed, perceptual reasoning or symptom severity in either medial or lateral Glx concentrations
Ohrmann et al. (2007)	chronic and FEP SZ patients vs. HC; correlations with neuropsychological performance measures	3.4 × 3.4 × 3.4 cm^3^ voxel in left DLPFC	20 chronic medicated patients; 15 FEP neuroleptic-naive patients; 20 healthy controls	1.5 T; STEAM sequence	Glx	5	Auditory Verbal Learning Test (AVLT), WAIS-R, figural learning task, modified WCST, reasoning task (measure of fluid intelligence); Attention Test Battery (TAP, Go-NoGo and Divided Attention)	positive and negative symptom, verbal and non-verbal learning and memory, IQ, attentional set shifting, selective and divided attention	*positive correlation in chronic patients*: Glx associated with AVLT **immediate recall score**; No sign. correlations with other assessment scores reported
							PANSS, Clinical Global Impression Scale (CGI)		

MRS studies reporting correlations between combined glutamate + glutamine (Glx) concentrations in frontal brain areas of chronic schizophrenia (SZ) patients and cognitive functions or severity of other symptoms; BPRS: Brief Psychiatric Rating Scale; PANSS: Positive and Negative Syndrome Scale; RBANS: Repeatable Battery for the Assessment of Neuropsychological Status; WM: working memory; AP: antipsychotics; FEP: first-episode patients; DLPFC: dorsolateral prefrontal cortex.

**Table 12:** First-episode patients: Correlations between GABA and cognitive functions or symptom severity.

Authors	Study design	Voxel size and location	Sample and medication	Field strength; spectroscopy scanning sequence	Neurometabolites	modified Newcastle-Ottawa score	Assessment tools	Investigated functions/ symptoms	Results
Wang et al. (2019)	FEP vs HC; correlations of neurometabolite levels with attentional, memory and executive functions	2 × 3 × 2 cm^3^ voxel in medial frontal areas; 2 × 2.5 × 2 cm^3^ voxel in left DLPFC	81 medicated FEP; 91 HC	7 T; STEAM sequence	GABA	6	neuropsychological test battery covering 6 cognitive domains	processing speed; attention/working memory; verbal memory; visual memory; ideational fluency; executive function	GABA correlated with executive functions in DLPFC in HC but not in patients
Reid et al. (2019)	FEP vs. HC; GABA correlations with neuropsychological measures	2.7 × 2.0 × 1.0 cm^3^ voxel in medial frontal areas	21 FEP; 21 HC	7 T; STEAM sequence	GABA	6	RBANS	General cognitive functions; specific focus on memory and language	**RBANS** total score: negative correlation with GABA in patients, but not HC; negative correlations of GABA with **immediate memory and language** subscales
Goto et al. (2009)	Patients within 6 months of disease onset; correlations with cognitive control measures and symptom severity	3 × 3 × 3 cm^3^ in medial frontal cortex	18 FEP patients	3 T; single voxel MEGA-PRESS sequence	GABA	6	WCST; PANSS	perseveration (WCST); positive and negative symptoms (PANSS)	trend towards neg. correlation between frontal GABA levels and better WCST **perseverative error** score

MRS studies reporting correlations between GABA concentrations in frontal brain areas of first-episode patients (FEP) and cognitive functions or severity of other symptoms; RBANS: Repeatable Battery for the Assessment of Neuropsychological Status; BPRS: Brief Psychiatric Rating Scale; PANSS: Positive and Negative Syndrome Scale; WCST: Wisconsin Card Sorting Test; WM: working memory; HC: healthy control participants; DLPFC: dorsolateral prefrontal cortex.

**Table 13:** First-episode patients: Correlations between Glutamate (Glu), Glutamine (Gln) or Glx and cognitive functions or symptom severity.

Authors	Study design	Voxel size and location	Sample and medication	Field strength; spectroscopy scanning sequence	Neurometabolites	modified Newcastle-Ottawa score	Assessment tools	Investigated functions/ symptoms	Results
Wang et al. (2019)	FEP vs HC; correlations of neurometabolite levels with attentional, memory and executive functions	2 × 3 × 2 cm^3^ voxel in medial frontal areas; 2 × 2.5 × 2 cm^3^ voxel in left DLPFC	81 medicated FEP; 91 HC	7 T; STEAM sequence	Glu	6	neuropsychological test battery covering 6 cognitive domains	processing speed; attention/working memory; verbal memory; visual memory;ideational fluency; executive function	*positive correlations:* between Glu and **verbal memory** performance in medial frontal areas in patients, but not in HC; positive correlations between Glu and **visual memory** performance in left DLPFC in patients but not in HC;
					Gln				
Reid et al. (2019)	FEP vs. HC; GABA correlations with neuropsychological measures	2.7 × 2.0 × 1.0 cm^3^ voxel in medial frontal areas	21 FEP; 21 HC	7 T; STEAM sequence	Glu	6	RBANS	General cognitive functions; specific focus on memory and language	No correlation with Gln or Glu in patients
					Gln				
Dempster et al. (2020)	FEP vs. HC, measuring treatment response; correlations with socio-occupational functioning	2 × 2 × 2 cm^3^ voxel in medial frontal areas	36 FEP (minimal treatment);27 HC	7 T; semi-LASER sequence	Glu	6	Social and Occupational Functioning Assessment Scale (SOFAS)	social and occupational functioning	*negative correlation:* higher Glu levels predicted **lower SOFAS** scores
Li et al. (2020)	Longitudinal design: drug-naïve FEP scanned at baseline and after 8 weeks of risperidone treatment vs. HC; correlations with negative symptom severity	medial frontal areas	35 drug-naïve first-episode patients; 40 HC	3 T;PRESS sequence	Glu, Glu/Cr + PCr (total creatine)	6	PANSS	negative symptoms as measured by PANSS	*negative correlation:* lower levels of Glu and Glu/Cr + PCr associated with more severe **negative symptoms**; after controlling for age, only the association between Glu/Cr + PCr remained significant
Bartolomeo et al. (2019)	FEP vs. HC; 31 FEP and 16 HC received additional EEG assessment; correlations with EEG measures and symptom severity	2 × 2 × 2 cm^3^ voxel in medial frontal areas	34 FEP participants; 19 HC	3 T; single voxel PRESS sequence	Glx	6	Electrophysiology: Mismatch negativity (MMN); auditory steady response (ASSR) power to 40 Hz stimulation; PANSS, BACS	impaired auditory processing (EEG), cognitive processing (BACS), positive, negative and disorganised thought symptoms (PANSS)	No sign. correlation
Jauhar et al. (2018)	FEP: correlation with negative symptoms (and positive symptoms)	2 × 2 × 2 cm^3^ voxel in medial frontal areas	28 FEP; 20 HC	3 T; PRESS	Glu	6	PANSS: positive and negative scores	positive and negative symptoms	no significant correlation between Glu levels and negative symptom scores; neg. correlation with positive symptoms scores (more Glu associated with fewer positive symptoms)
Olbrich et al. (2008)	FEP vs. HC; correlations with symptom severity	2 × 2 × 2 cm^3^ voxel in left DLPFC	9 medicated FEP; 32 HC	2 T; PRESS sequence	Glu	6	BPRS, Scale for the Assessment of Negative Symptoms (SANS)	positive and negative symptoms	*negative correlation:* higher Glu levels associated with less severe symptoms
					Gln				
Ohrmann et al. (2007)	chronic and FEP patients and HC	3.4 × 3.4 × 3.4 cm^3^ voxel in left DLPFC voxel	15 first-episode neuroleptic-naive patients;20 chronic patients; 20 HC	1.5 T	Glx	5	Auditory Verbal Learning Task	Memory performance	No sign. correlation between AVLT immediate recall scores and Glx in FEP (only in chronic patients)
				STEAM					

MRS studies reporting correlations between Glu, Gln, Glx concentrations in frontal brain areas of first-episode patients (FEP) and cognitive functions or severity of other symptoms; RBANS: Repeatable Battery for the Assessment of Neuropsychological Status; BPRS: Brief Psychiatric Rating Scale; PANSS: Positive and Negative Syndrome Scale; WM: working memory; HC: healthy control participants; DLPFC: dorsolateral prefrontal cortex.

### 3.2 Primary and secondary outcomes

#### 3.2.1 Neurometabolite differences in individuals with chronic schizophrenia

We reviewed studies that investigated GABA, Glu, Gln and Glx modulations in frontal brain areas in chronic schizophrenia patients compared to a healthy control group (Table 1, Table 2, Table 3, Table 4, respectively). Notably, chronic patients had often received a stable treatment of antipsychotics prior to the study which may play a role in metabolite concentrations.

For GABA, the findings were mixed between a GABA reduction in schizophrenia patients (4 studies) and no difference to healthy controls (5 studies; Table 1). The GABA study using the highest magnet field strengths (Marsman et al., 2014), and therefore having higher sensitivity for GABA modulations (Terpstra et al., 2016), showed indeed a **GABA reduction** in medial frontal areas. Other studies with lower field strength tended to find GABA level reductions particularly in older patients. All studies that reported a GABA reduction used a voxel location in the **medial frontal cortex**, and half of these studies reported GABA levels as ratio with Cr. One study (Marenco et al., 2016) demonstrated a GABA reduction only for patients treated with antipsychotics, but not for untreated patients, whereas the only study that reported a GABA increase (Kegeles et al., 2012), only found this effect in unmedicated patients.

Similarly, the results for **Glu** modulations in frontal brain areas of patients with chronic schizophrenia are mixed (Table 2). Only two studies, using a low field strength of 2 T, found a Glu increase in patients, whereas 7 studies reported a Glu level reduction in patients, and 8 studies reported no difference in Glu levels. At least two studies (Shukla et al., 2019; Chiappelli et al., 2015) mentioned a significant relationship between Glu levels in medial frontal brain areas and age of the patients with older patients showing lower Glu levels. It might be noteworthy that three studies reporting a reduction in Glu levels used a STEAM scanning sequence, while only one study that did not find a modulation in Glu, used a STEAM sequence and this was the study with the lowest field strength. Most studies that did not report a Glu modulation in patients employed variations of PRESS scanning sequences.

Only a few studies reported **Gln** levels in chronic schizophrenia patients (Table 3). Two studies that employed higher field strength (7T or 4T) in their MRS measurements reported a **Gln reduction** in medial frontal brain areas in patients (Kumar et al., 2020; Théberge et al., 2003). Four studies with lower field strengths magnets reported an **increase in Gln** (Bustillo et al., 2014; Tebartz Van Elst et al., 2005; Stanley et al., 1996; Rüsch et al., 2008). Notably, most of these studies reporting an increase used a voxel location in the left DLPFC. Bustillo et al. (2014) found a Gln level increase with age. Two studies did not find any Gln modulations in medial frontal voxels (Rowland et al., 2016b; Shirayama et al., 2010). Overall, the evidence for Gln modulations in chronic SZ patient is currently rather weak, but there might be a tendency for decreased Gln levels in medial frontal brain areas and a tendency towards an increase of Gln levels in left lateral frontal areas.

The largest study that investigated **Glx** modulations (see Table 4) in frontal brain areas in chronic schizophrenia patients reported **reduced Glx** levels in their sample (Bustillo et al., 2017). Overall, 9 studies demonstrated reduced Glx levels in patients compared to a healthy control group (Bustillo et al., 2017; Ćurčić-Blake et al., 2017; Liemburg et al., 2016; Cadena et al., 2018; Natsubori et al., 2014; Hugdahl et al., 2015; Rowland, Kontson et al., 2013; Ohrmann et al., 2007, 2005), 10 studies did not find significant differences in Glx (Kraguljac et al., 2018; Chiappelli et al., 2018; Reid et al., 2010; Shah et al., 2020; Kegeles et al., 2012: for medicated patients; Coughlin et al., 2015; Rowland et al., 2009; Goldstein et al., 2015; Ota et al., 2007; Szulc et al., 2013), and 2 studies reported Glx increases in frontal brain areas, although Kegeles et al. (2012) reported a Glx increase only in unmedicated patients. Additionally, Hjelmervik et al. (2020) reported both a Glx increase in patients that were less affected by auditory hallucinations, while the group of patients that was more affected by auditory hallucinations showed a Glx reduction in medial frontal brain areas. Liemburg et al. (2016) found a negative correlation with illness duration in Glx levels of chronic patients.

#### 3.2.2 Neurometabolite differences in individuals with first-episode schizophrenia

For first-episode (FEP) schizophrenia patients, there were seven studies that have investigated changes in **GABA** levels in frontal brain areas (Table 5). Cen et al. (2020) reported a GABA increase in drug-naïve FEP in ventromedial brain areas. De la Fuente-Sandoval et al. (2017) found an increase in GABA levels only in unmedicated patients, but no difference to healthy controls in medicated patients. Thus, both results showing a **GABA increase** are associated with **unmedicated patients**. Three studies reported **reduced GABA** levels in medial frontal brain areas (Wang et al., 2019, 2016; Bojesen et al., 2020). Bojesen et al. (2020) investigated treatment responses in FEP and found a GABA decrease in treatment non-responders only. Two studies (Reid et al., 2019; Goto et al., 2010) did not find any difference in frontal GABA levels.

Most studies that reported **Glu, Gln or Glx** levels in frontal brain areas showed **no difference** between FEP and healthy controls (7 studies (Table 6), 5 studies (Table 7), and 7 studies (Table 8), respectively). Three studies reported a Glu reduction (Reid et al., 2019; Wang et al., 2019; Bojesen et al., 2020) in medial frontal areas. In contrast, Olbrich et al. (2008) reported a Glu increase at 2 T in left lateral frontal areas.

For Glx, 2 studies (Bartolomeo et al., 2019; Ohrmann et al., 2007) reported an increase in medial or left frontolateral areas in FEP patients, whereas one study found a Glx decrease in FEP (Natsubori et al., 2014).

Overall, there is a lack of studies with larger sample sizes in first-episode patients.

Four of the included studies used cohorts of patients from both the chronic and first episodic phases of illness, allowing a direct comparison for metabolic levels without confounding variabilities in research methods. Ohrmann et al. (2007) and Ohrmann et al. (2005) both used magnet strength of 1.5 T and reported that the Glx levels of chronic patients were significantly lower than that of controls and FEP in the DLPFC, however measures between FEP and controls were not significantly different. Stanley et al. (1996) found the only significant difference between groups was an increased level of Gln in chronic patients when compared with controls, however the efficacy of Gln measures at 1.5 T is debated. Natsubori et al. (2014) additionally included familial relatives of patients to index the metabolite levels of those at ultra-high risk (UHR). Comparisons yielded a significant effect of diagnosis duration with an increase in medial frontal Glx through the groups (chronic patients exhibiting the highest levels).

#### 3.2.3 Chronic patients: correlations between frontal neurometabolite concentrations and cognitive functions

Correlations between neurometabolite concentrations and cognitive functions in chronic SZ patients are summarised in Table 9, Table 10.

##### 3.2.3.1 Working memory

Relationships between frontal neurometabolite concentrations and cognitive functions have not been studied systematically yet. However, 10 studies have investigated working memory performance in association with neurometabolites in frontal brain areas. Out of these 10 studies, two reported positive correlations between **medial frontal GABA** concentrations and WM performance (Rowland et al., 2016a; Rowland et al., 2016b), i.e. higher medial frontal GABA concentrations were associated with better WM performance. Ohrmann et al. (2007) found frontolateral Glx concentrations to be positively associated with improved immediate recall in the Auditory Verbal Learning Task (AVLT), and Kaminski et al. (2020) reported a positive correlation between the WM-related BOLD response in the **left dorsolateral prefrontal cortex (DLPFC)** and **Glu** concentrations in this brain area.

In contrast, two studies showed negative correlations between WM performance and the frontomedial GABA/Cr ratio (Marsman et al., 2014) or the frontomedial Gln/Glu ratio (Shirayama et al., 2010). Four studies did not find a significant relationship between frontal GABA, Glu or Glx concentrations and WM performance (Kegeles et al., 2012; Rowland et al., 2013; Chiappelli et al., 2015).

##### 3.2.3.2 Processing speed

Two studies reported a **positive** correlation between processing speed and **medial frontal GABA** concentrations in chronic schizophrenia patients (Rowland et al., 2016b; Rowland et al., 2013), while Rowland et al. (2016a) did not find a significant correlation with medial GABA. Frontal Glu (Chiappelli et al., 2015; Shirayama et al., 2010) or Glx concentrations (Rowland et al., 2013; Ohrmann et al., 2008) do not appear to be related to processing speed.

##### 3.2.3.3 Mismatch negativity or prediction errors

Rowland et al. (2016b) investigated the mismatch negativity (MMN), which is an electrophysiological signal that reflects the detection of deviations from predicted events. In chronic schizophrenia patients, they found that larger MMN amplitudes are associated with **higher GABA** and **Glu** concentrations in **medial** frontal brain areas.

##### 3.2.3.4 Set shifting

Ohrmann et al. (2008) reported a positive correlation between the learning potential in the Wisconsin Card Sorting Test and Glx concentration in medial frontal, but not lateral frontal areas. Rüsch et al. (2008) and Shirayama et al. (2010) investigated frontal Glu or Gln levels or the Gln/Glu ratio in relation to WCST performance but did not find a significant correlation.

##### 3.2.3.5 Other cognitive measures

Bustillo et al. (2011) reported a positive correlation between a general cognitive factor, derived from a factor analysis across a range of different neuropsychological tests, and Glx concentrations in patients.

Two studies investigated perceptual reasoning in chronic schizophrenia patients: Marsman et al. (2014) found a negative correlation with the GABA/Cr ratio in medial frontal areas, i.e. better perceptual reasoning performance was associated with a lower GABA/Cr ratio in patients (but not in controls), while Ohrmann et al. (2008) investigated Glx concentrations, but did not find any significant correlation with perceptual reasoning functions.

Marsman et al. (2014) additionally reported negative correlations between the medial GABA/Cr ratio and both IQ scores and verbal comprehension abilities. Tebartz van Elst et al. (2005) showed a negative correlation between Glu concentrations in the left DLPFC and psychosocial functioning.

Interference effects (e.g. in a Stroop task) did not correlate with frontomedial Gln/Glu or Glx/Cr ratios (Shirayama et al., 2010; Reid et al., 2010).

#### 3.2.4 Chronic patients: correlations between frontal neurometabolite concentrations and symptom severity

Studies that have investigated correlations between GABA levels in medial frontal brain areas and symptom severity in chronic schizophrenia patients did not find a significant relationship (Marsman et al., 2014; Rowland et al., 2016a, b; Kegeles et al., 2012; Rowland et al., 2013; Table 9), while the only study that investigated GABA + in the left **DLPFC** (Xiang et al., 2019) did report a positive correlation with the PANSS total score, indicating that higher GABA + levels are associated with more severe symptoms.

For Glu concentrations, no study with a voxel location in **medial frontal** areas did report significant correlations between Glu and symptom scores (Chiappelli et al., 2015; Rowland et al., 2016b; Shirayama et al., 2010).

There is mixed evidence regarding symptom severity correlations with frontal Glx concentrations. Hugdahl et al. (2015) reported a positive correlation between Glx in lateral frontal areas and positive symptoms (hallucinations). Reid et al. (2010) demonstrated a negative correlation between medial Glx/Cr ratios and negative symptoms, with lower ratios predicting more negative symptoms. On the other hand, Xiang et al. (2019) showed a positive correlation between left DLPFC Glx levels and negative symptom severity. Seven studies did not find any significant correlations between Glx measures and symptom severity (Goldstein et al., 2015; Liemburg et al., 2016; Rowland et al., 2009; Ohrmann et al., 2005, 2008; Kegeles et al., 2012; Rowland et al., 2013). Just one study (Bustillo et al., 2014) investigated Gln concentrations in association with symptom scores and found a positive correlation between medial Gln levels and positive symptoms. Kumar et al. (2020) found that patients with residual schizophrenia showed marked reductions in Glu.

#### 3.2.5 First-episode patients: correlations between frontal neurometabolite concentrations and cognitive functions

Only very few studies have investigated the relationship between frontal neurometabolite levels and cognitive functions in first-episode patients (FEP) so far. The most comprehensive studies (Reid et al., 2019; Wang et al., 2019) in this research area were conducted at 7 T. Reid et al. (2019) investigated GABA, Glu and Gln in medial frontal brain areas in association with different subscale scores of the Repeatable Battery for the Assessment of Neuropsychological Status (RBANS). The authors reported **negative** correlations between medial frontal **GABA** levels and the **memory and language scores** of the RBANS as well as the overall RBANS score, i.e. lower GABA levels were associated with better performance in the RBANS. They did not find similar correlations for Glu or Gln levels. In contrast, Wang et al. (2019) reported **positive** correlations between **Glu** levels in **medial** frontal areas of FEP patients and **verbal memory**, and between **left DLPFC Glu** levels and **visual memory** scores. One other study (Ohrmann et al., 2007) investigated memory performance in the context of Glx levels within the left DLPFC but did not find a significant correlation.

Wang et al. (2019) did not report any significant relationships between either medial or lateral frontal neurometabolites and processing speed or executive functions.

Dempster et al. (2020) investigated social and occupational functioning (SOFAS) in FEP patients and reported a negative correlation with frontomedial Glu levels, i.e. higher Glu concentrations were associated with lower social and occupational functioning scores.

#### 3.2.6 First-episode patients: correlations between frontal neurometabolite concentrations and symptom severity

Two studies (Li et al., 2020; Olbrich et al., 2008) reported negative correlations between frontal Glu levels and negative symptom severity with lower Glu levels being associated with more negative symptoms. In contrast, Jauhar et al. (2018) did not find a significant correlation between medial frontal Glu levels and negative symptoms, but a negative correlation between Glu and positive symptom severity, i.e. lower Glu concentrations predicted more positive symptoms.

Frontal Gln and Glx levels were not significant associated with symptom severity in FEP.

Both Olbrich et al. (2008) and Li et al. (2020) found significant negative correlation between frontal Glu levels in FEP patients, and their scores on BACS and PANNS-N respectively. This effect was not replicated by Bartolomeo et al. (2019) who also failed to find a significant relationship with the mismatch negativity results. Overall, the evidence for correlations between frontal neurometabolite levels and symptom severity in FEP patients is rather inconclusive.

## 4 Discussion

Several studies have investigated general differences in GABA, glutamate (Glu), glutamine (Gln) and Glx levels in frontal lobe areas of both chronic and first-episode schizophrenia patients. While the results across entire populations remain varied, there appears to greater homogeneity when comparing chronic and first-episode patients separately.

Evidence for correlations between cognitive control functions and GABA, Glu, Gln and Glx neurometabolite levels in frontal brain areas is still limited, however, more recently several studies have been added to this line of research, thus, some patterns seem to emerge, especially in chronic SZ patients. Only very few studies have investigated these relationships in first-episode patients. We will first discuss overall differences in frontal metabolite levels between patients and healthy control individuals and then turn to studies that have investigated correlations between frontal neurometabolites and symptoms or cognitive control functions, respectively.

### 4.1 General metabolite differences between SZ patients and healthy control groups in frontal brain areas

In general, a lot of variability can be found when comparing frontal GABA, Glu, Gln and Glx levels between individuals with schizophrenia and healthy control groups. GABA studies showed reduced medial frontal metabolite levels in medically treated or older chronic patients or when GABA was investigated with ultra-high field MRS (7 T) perhaps indicating that prior inconsistencies may be due to technical limitations (Marsman et al., 2014). However, several studies did not find a difference in frontal GABA concentrations between SZ patients and their control group. The study quality was comparable between those studies reporting reduced GABA levels and those studies that did not find a difference between patients and control participants.

Glu levels also demonstrated similar disparities, with studies reporting either no significant difference or a Glu reduction in frontomedial regions of chronic patients. In FEP patients, the majority of studies did not find significant differences in Glu levels, but two studies that employed higher field strengths (Reid et al., 2019; Wang et al., 2019) demonstrated reduced Glu level in frontomedial areas. Therefore, the Glu results seem to be similar for chronic and FEP patients.

Studies that reported frontal Gln levels in chronic patients found reduced Gln concentrations when employing higher field strengths, while studies conducted at lower field strengths did not report Gln differences or even an increase in Gln. However, Bustillo et al. (2014) reported a positive correlation between Gln levels and age in chronic patients. Therefore, the variability in results could be due to different age ranges of patients, but also due to different field strengths as suggested by Marsman et al. (2014). For FEP patients, the overall results suggest no difference in frontal Gln between patients and control groups.

In addition to separately reported Gln and Glu measurements, studies at a lower field strength reported combined measurements as Glx. With this combined metabolic measurement, slightly more studies reported reduced levels of Glx, especially in FEP patients, perhaps indicating that variance in Glu and Gln measurements may reflect an interaction of the two metabolites and how they are affected by schizophrenia (Bustillo et al., 2017).

A potential factor that attributed to the variance in results, is the use of antipsychotics (AP) in patients. This is particularly prominent within the chronic cohort of patients, as they have been receiving treatment for the condition longer than the FEP patients. Long term use of AP has been shown to have mixed results in the treatment of schizophrenia and can also change frontal metabolite measurements making comparisons between unmedicated and medicated patients questionable (Harrow and Jobe, 2013). While significant differences between sexes have not been noted for glutamate levels, there have been results that indicate that age plays a large role in glutamate levels in patients (Shukla et al., 2019; Chiappelli et al., 2015). Studies have shown a significant change in glutamatergic action as a function of age, in tandem with a loss of NAA which serves as a marker for neuronal viability (Urenjak et al., 1992). Global changes in glutamate levels have been observed across the whole brain. Segovia et al. (2001) suggest that inconsistencies in metabolic results may be due to compensatory release of glutamate in response to a global reduction. It is suggested that a better measure would be to evaluate the quantity and quality of NMDA receptors as glutamate measures could reflect glutamate release, or ineffectual glutamate uptake. As there seems to be a significant change to the glutamatergic system with age, it becomes difficult to make accurate comparisons between chronic and FEP patients as age almost always represents a confounding factor. However, when controlled for age several studies reported in the tables above did still find significant deviations from HC. This may indicate an interaction between schizophrenia and the natural deterioration of the glutamatergic system. Squires-Wheeler et al. (1993) indeed reported a loss of Glu neurons in medial frontal and other brain areas in post-mortem brains of schizophrenia patients.

This review revealed preliminary evidence for associations between neurometabolites in frontal brain areas, particularly GABA and Glu levels, and cognitive control functions. Metabolite deviations associated with impairments in cognitive control functions have been linked with a number of mental health conditions, including attention deficit hyperactivity disorder (ADHD), mood disorders (Reddy-Thootkur et al., 2020) and anxiety (Naaijen et al., 2018; Morgenroth et al., 2019). Occasionally, frontal differences in functional imaging have been associated with deviations in metabolic measurements, however, measured independently from improvements in cognitive control (Basten et al., 2012). However, the reported findings show considerable variability. One reason for this variability could be that cognitive functions are typically associated with different areas within the frontal lobes (cf. Ullsperger et al., 2014; Braver et al., 2009; Brosnan and Wiegand, 2017). In contrast, the reported MRS voxel sizes are relatively large (cf. Michou et al., 2015), potentially comprising several different functional areas within the frontal lobes. Small variations in voxel positions across studies could potentially lead to different results as different functional areas might have been covered, thereby increasing variability in results across studies.

### 4.2 Associations between cognitive functions and frontal metabolite levels

There are established associations between frontal brain regions and cognitive control functions (e.g. Ullsperger et al., 2014), and the glutamatergic system has further been associated with fronto-striatal projections which are crucial for the implementation of cognitive control (Naaijen et al., 2018). These frontal projections have been shown to modulate task-specific activity in posterior regions of the brain and implement behavioural inhibition crucial to the effective action of behaviour through GABAergic interneurons.

In the context of goal-directed behaviour, WM is relevant for goal maintenance (Barch and Ceaser, 2012; Friedman and Robbins, 2021). Several studies have investigated the relationship between working memory (WM) performance and neurometabolites in frontal brain areas. In chronic SZ patients, WM performance seems to be positively correlated with medial frontal GABA levels and frontolateral Glu or Glx concentrations (Rowland et al., 2016a, b; Kaminski et al., 2020; Ohrmann et al., 2007). However, those studies that quantified GABA or Gln as ratio to other metabolites reported negative correlations instead (Marsman et al., 2014; Shirayama et al., 2010). For FEP patients, more evidence is required. Recent ultra-high field MRS studies (Wang, Pradhan et al., 2019; Reid et al., 2019) suggest potential associations between WM performance and GABA, Glu and GSH levels in this group of patients, but verbal and visual memory performance might need to be investigated separately in future studies as in Wang et al. (2019).

Processing speed might influence internal monitoring processes as the timing of incoming sensory information and internally generated predictions could be critical to detect conflict or suboptimal action outcomes. Processing speed is consistently reduced in individuals with schizophrenia (e.g. Habtewold et al., 2020). Studies reviewed here suggest that medial frontal GABA levels predict processing speed (Rowland et al., 2016b; Rowland et al., 2013), with higher GABA levels being associated with higher processing speed in chronic schizophrenia patients. However, there were no significant associations in with processing speed in FEP patients. The association between GABA levels and processing speed in chronic patients is in line with the finding that a genetic variation in the CADM2 gene is related to individual differences in information processing speed in healthy individuals. This genetic variant is expressed in the cingulate cortex and the protein that is encoded by CADM2 plays a role in glutamate signalling and GABA transport (Ibrahim-Verbaas et al., 2016).

The mismatch negativity (MMN), which is related to the processing of prediction errors (e.g. den Ouden et al., 2012), showed a positive correlation with medial frontal GABA and Glu levels in chronic patients (Rowland et al., 2016b), but not in FEP patients (Bartolomeo et al., 2019). Previously, GABA-related polymorphisms have been associated with modulations in the processing of prediction errors (Baetu et al., 2018), supporting the results by Rowland et al. (2016b). However, the evidence for this relationship is currently very limited and more studies are required to further investigate the role of medial frontal GABA and Glu concentrations in prediction errors. Similarly, the evidence for other potential relationships between frontal metabolite levels and cognitive performance in schizophrenia patients is not very robust yet.

Overall, frontomedial GABA levels and frontomedial and -lateral Glu levels seem to be associated with different aspects of cognitive control functions in schizophrenia patients. A limitation of many articles reporting correlations between cognitive functions and neurometabolites is that the difference in correlations in patient groups and in corresponding correlations in a healthy control group are often not reported. There are also inconsistencies in that some studies report correlations across both patients and control group participants while other studies calculate separate correlations for patients and control participants. A more consistent approach in reporting these correlations would be desirable.

### 4.3 Associations between schizophrenia symptoms and frontal metabolite levels

The majority of studies did not find a significant relationship between the degree of schizophrenia symptoms and metabolite levels in frontal brain areas. The review revealed that the overall score of symptom severity scales (e.g. BPRS or PANSS) is not well suited to predict frontal metabolite levels (but see Xiang et al., 2019). Though, several studies showed significant associations between different subscales (e.g. measuring just positive or negative symptoms) and metabolite levels, but the results represented a mix of positive and negative correlations in chronic SZ patients. Negative symptoms have been shown to be associated with frontal Glx levels in chronic SZ patients (negative correlation in medial areas and a positive correlation in frontolateral areas), and with Glu levels in FEP patients (negative correlations; Li et al., 2020; Olbrich et al., 2008; but see Jauhar et al., 2018).

### 4.4 Conclusions and future directions

GABA and Glu concentrations seem to be relevant neurometabolites that are altered in individuals with schizophrenia. GABA and Glu levels in frontal brain areas also seem to be associated with performance in cognitive control functions. However, there is considerable variability in the results across studies. Heterogeneity in the clinical presentation of schizophrenia is a key factor which contributes to this variability. Recruiting homogeneous patient groups is difficult, and therefore, accurate reporting of clinical features in publications is important as it will aid our understanding of the link between symptoms, cognitive/socio-occupational functioning and neurometabolite alterations. In patients with chronic schizophrenia, in addition to a cross-section snapshot of symptoms, a method to assess and document the lifetime history of psychotic and other symptoms could prove to be very valuable.

Medication use is another related, important factor. The effect of current medication use on MRS findings is typically accounted for by most studies, but the impact of long-term medication use on neurometabolite levels is still not fully understood. A systematic review of longitudinal studies by Egerton et al. (2017) reported a reduction in mean Glx levels following antipsychotic treatment in schizophrenia, however this included only 8 studies as this type of data is currently limited. More longitudinal studies are needed to fully explore this complex issue of changes related to medication use and to distinguish them from disease-related changes.

MRS studies at higher field strengths are recommended, particularly for studies measuring glutamate as it is difficult to separate glutamate from glutamine at lower field strengths. Similarly, GABA can be measure more reliably at ultra-high field strengths (Terpstra et al., 2016). Importantly, a precise description of the anatomical position of the MRS voxels could aid with the interpretation of the findings in association with cognitive functions as different cognitive control functions have been associated with different neuroanatomical areas within the frontal lobes (e.g. Friedman and Robbins, 2021; Ullsperger et al., 2014). Standardised data acquisition methods and analysis pipelines could also be helpful with directly comparing results from studies. A few studies have conducted functional MRS experiments (e.g., Kaminski et al., 2020) where metabolite levels are quantified at baseline and after participants have completed a task that activates the brain area of interest. These kinds of studies could lead to more precise insights into the relationship between neurometabolite levels and cognitive functions. Similarly, multi-modal study designs e.g., combining MRS with MEG or TMS, could also be extremely useful as they can provide important complementary information (Kempton and McGuire, 2015). Additionally, a greater focus of attention toward the role of GSH could provide greater insight into this research area. Some studies (e.g. Dempster et al., 2020; Kumar et al., 2020) within this systematic review collected GSH data and reported correlations with cognitive functions (Wang et al., 2019), but given that it was not a primary research focus at the outset of the review we did not comprehensively search for it. Overall, more systematic studies are required to further establish the association between cognitive functions and neurometabolite levels and add to the evidence regarding other neurometabolites.

## Declaration of Competing Interest

The authors report no declarations of interest.

## Figures and Tables

**Table:** 

AUTHOR	SELECTION				COMPARABILITY	TOTAL
	1. Case Definition	2. Representativeness	3. Selection	4. Definition	1. Design	
Aoyama et al. (2011)	*	*	*	*	**	6
Bartolomeo et al. (2019)	*	*	*	*	**	6
Bojesen et al. (2020)	*	*	*	*	**	6
Bustillo et al. (2014)	*	*	*	*	**	6
Bustillo et al. (2017)	*	*	*	*	**	6
Bustillo et al. (2010)	*	x	*	*	**	5
Cadena et al. (2018)	*	*	*	*	**	6
Cen et al. (2020)	*	*	*	*	**	6
Chang et al. (2007)	*	X	*	*	**	5
Chiappelli et al. (2018)	*	*	*	*	**	6
Chiappelli et al. (2015)	*	*	*	*	**	6
Coughlin et al. (2015)	*	*	*	*	**	6
Ćurčić-Blake et al. (2017)	*	*	*	*	**	6
De le Fuente-Sandoval et al. (2017)	*	*	*	*	**	6
Dempster et al. (2020)	*	*	*	*	**	6
Gallinat et al. (2016)	*	*	*	*	**	6
Galinska et al. (2009)	*	*	*	*	**	6
Girgis et al. (2019)	*	X	*	*	**	5
Goldstein et al. (2015)	*	*	*	*	**	6
Goto et al. (2009)	*	*	*	*	**	6
Goto et al. (2010)	*	*	*	*	**	6
Goto et al. (2012)	*	*	*	*	**	6
Hjelmervik et al. (2020)	*	*	*	*	**	6
Hugdahl et al. (2015)	*	*	*	*	**	6
Jauhar et al. (2018)	*	*	*	*	**	6
Kaminski et al. (2020)	*	*	*	*	**	6
Kegeles et al. (2012)	*	X	*	*	**	5
Kraguljac et al. (2018)	*	*	*	*	**	6
Kumar et al. (2020)	*	*	*	*	**	6
Li et al. (2020)	*	*	*	*	**	6
Liemburg et al. (2016)	*	*	*	*	**	6
Marenco et al. (2016)	*	*	*	*	**	6
Marsman et al. (2014)	*	*	*	*	**	6
Natsubori et al. (2014)	*	*	*	*	**	6
Ohrmann et al. (2005)	*	*	*	*	X*	5
Ohrmann et al. (2007)	*	X	*	*	**	5
Ohrmann et al. (2008)	*	*	*	*	**	6
Olbrich et al. (2008)	*	*	*	*	**	6
Ota et al. (2012)	*	*	*	*	**	6
Reid et al. (2019)	*	*	*	*	**	6
Reid et al. (2010)	*	*	*	*	**	6
Rowland et al. (2013)	*	*	*	*	**	6
Rowland et al. (2016a)	*	*	*	*	X*	5
Rowland et al. (2009)	*	*	*	*	**	6
Rowland et al. (2016b)	*	X	*	*	X*	4
Rüsch et al. (2008)	*	*	*	*	**	6
Shah et al. (2020)	*	*	*	*	**	6
Shirayama et al. (2010)	*	*	*	*	**	6
Shukla et al. (2019)	*	*	*	*	**	6
Stanley et al. (1996)	*	*	*	*	**	6
Szulc et al. (2011)	*	*	*	*	**	6
Szulc et al. (2013)	*	X	*	*	**	5
Tayoshi et al. (2010)	*	*	*	*	**	6
Tebartz van Elst et al. (2005)	*	*	*	*	**	6
Théberge et al. (2003)	*	*	*	*	**	6
Théberge et al. (2002)	*	*	*	*	**	6
Wang et al. (2019)	*	*	*	*	**	6
Wang et al. (2016)	*	*	*	*	**	6
Xiang et al. (2019)	*	*	*	*	**	6

## Appendix A

### MODIFIED NEWCASTLE-OTTAWA SCALE

Below is a description of each of the criteria that study quality was assessed on before being entered into the systematic review. A study must meet the required quality to be awarded a star for each criterion. A maximum of one star was awarded for each criterion in the Selection category, and a maximum of two stars in the Comparability category.

#### SELECTION

1 Is the case definition adequate?
  - Diagnosis of schizophrenia has more than one independent verification of disease (i.e. initial clinical diagnosis, and appraisal of symptoms during study) - 1 star (*)
2 Representativeness of the cases
  - Sample reflects all participants with appropriate diagnosis of schizophrenia in a given population (i.e. no exclusion based on gender / age demographic information). An exemption is made for exclusion based on diagnosis length (First-episodic diagnosis vs chronic illness duration). Continuous sample of participants that is representative of the entire patient population was used - 1 star (*)
  - No star: Non-random sampling of participants (i.e. use of a pre-selected group of patients who had indicated eagerness to participate in research)
3 Selection of control
  - Study presents details of population that healthy controls were taken from. Study must present details of matching process (e.g. age-matched, sex-matched, socio-economic status etc.) - 1 star (*)
4 Definition of controls / exclusionary criteria
  - Study must present adequate exclusion criteria for healthy control participants in the research. This must include at least: free from diagnosis from schizophrenia or other major psychiatric condition; free from alcohol dependency; free from illicit drug use; free from prescription drug use for psychiatric purposes - 1 star (*)

#### COMPARABILITY

1 Comparability of study on the basis of design of analysis
  - Definition of voxel size, dimensions, and location with reference to neurological anatomy is given to ensure that the prescribed area is comparable between studies in the literature - 1 star (*)
  - Description of magnetic resonance imaging procedure. This includes both magnetic field strength information (Tesla) and pulse sequencing information from the magnetic resonance imaging design (e.g. MEGA-PRESS) - 1 star (*)
